# Lymphocyte Non-Specific Function Detection Facilitating the Stratification of *Mycobacterium tuberculosis* Infection

**DOI:** 10.3389/fimmu.2021.641378

**Published:** 2021-04-19

**Authors:** Ying Luo, Ying Xue, Yimin Cai, Qun Lin, Guoxing Tang, Huijuan Song, Wei Liu, Liyan Mao, Xu Yuan, Yu Zhou, Weiyong Liu, Shiji Wu, Ziyong Sun, Feng Wang

**Affiliations:** ^1^ Department of Laboratory Medicine, Tongji Hospital, Tongji Medical College, Huazhong University of Science and Technology, Wuhan, China; ^2^ Department of Immunology, School of Basic Medicine, Tongji Medical College, Huazhong University of Science and Technology, Wuhan, China; ^3^ Department of Epidemiology and Biostatistics, Key Laboratory of Environmental Health of Ministry of Education, School of Public Health, Tongji Medical College, Huazhong University of Science and Technology, Wuhan, China; ^4^ Department of Laboratory Medicine, Zhejiang Provincial People’s Hospital, People’s Hospital of Hangzhou Medical College, Hangzhou, China

**Keywords:** tuberculosis, active tuberculosis, latent tuberculosis infection, diagnosis, model, lymphocyte non-specific function

## Abstract

**Background:**

Inadequate tuberculosis (TB) diagnostics, especially for discrimination between active TB (ATB) and latent TB infection (LTBI), are major hurdle in the reduction of the disease burden. The present study aims to investigate the role of lymphocyte non-specific function detection for TB diagnosis in clinical practice.

**Methods:**

A total of 208 participants including 49 ATB patients, 64 LTBI individuals, and 95 healthy controls were recruited at Tongji hospital from January 2019 to October 2020. All subjects were tested with lymphocyte non-specific function detection and T-SPOT assay.

**Results:**

Significantly positive correlation existed between lymphocyte non-specific function and phytohemagglutinin (PHA) spot number. CD4^+^ T cell non-specific function showed the potential for differentiating patients with negative T-SPOT results from those with positive T-SPOT results with an area under the curve (AUC) of 0.732 (95% CI, 0.572-0.893). The non-specific function of CD4^+^ T cells, CD8^+^ T cells, and NK cells was found significantly lower in ATB patients than in LTBI individuals. The AUCs presented by CD4^+^ T cell non-specific function, CD8^+^ T cell non-specific function, and NK cell non-specific function for discriminating ATB patients from LTBI individuals were 0.845 (95% CI, 0.767-0.925), 0.770 (95% CI, 0.683-0.857), and 0.691 (95% CI, 0.593-0.789), respectively. Application of multivariable logistic regression resulted in the combination of CD4^+^ T cell non-specific function, NK cell non-specific function, and culture filtrate protein-10 (CFP-10) spot number as the optimally diagnostic model for differentiating ATB from LTBI. The AUC of the model in distinguishing between ATB and LTBI was 0.939 (95% CI, 0.898-0.981). The sensitivity and specificity were 83.67% (95% CI, 70.96%-91.49%) and 90.63% (95% CI, 81.02%-95.63%) with the threshold as 0.57. Our established model showed superior performance to TB-specific antigen (TBAg)/PHA ratio in stratifying TB infection status.

**Conclusions:**

Lymphocyte non-specific function detection offers an attractive alternative to facilitate TB diagnosis. The three-index diagnostic model was proved to be a potent tool for the identification of different events involved in TB infection, which is helpful for the treatment and management of patients.

## Introduction

Tuberculosis (TB) is a major public issue caused by *Mycobacterium tuberculosis* (MTB) infection, with around 10 million cases and 1.4 million deaths in 2019 reported by World Health Organization (1). It is estimated that one-quarter population were during latent TB infection (LTBI) and 5-10% of these individuals would progress to active TB (ATB) during their life (2, 3). The stratification of TB infection is required for proposed TB control strategies that focus on timely treatment to reduce risk for disease progression in order to diminish MTB transmission (4). However, the current challenge still includes the lack of effective approach for discrimination between ATB and other status including LTBI. Novel and accurate diagnostic tests to identify active cases are urgently needed.

The diagnosis of ATB could be achieved by visualization of acid-fast bacilli by microscopy, mycobacterial culture, or molecular tests including GeneXpert MTB/RIF. Nevertheless, each approach has additional limitations, such as the poor sensitivity of microscopy, time-consuming of culture, the high cost of molecular tests (5). Even in the era of the GeneXpert MTB/RIF Ultra, challenge remains due to unsatisfactory sensitivity for clinical requirement (6, 7), which highlights the fact that better diagnostics might have to be achieved based on host factors rather than pathogen detection. However, the current use of blood-based available immunological tests including T-SPOT.TB (T-SPOT) and QuantiFERON-TB Gold In-Tube (QFT-GIT) was limited by their poor ability to reliably stratify ATB from LTBI especially in TB endemic areas (8, 9). Recent advances have been developed in blood signatures including transcriptome (10), proteome (11), genome (12), metabolome (13), cytokines (14, 15), and markers on immune cells (16, 17) for identifying ATB, raising hopes for translation into available assays. However, due to the fact that the application of these emerging methods has not been verified with sufficient repetition and large sample size, the real diagnostic utility under actual clinical conditions remains unclear (18). Besides, some tests require complicated procedures or expensive equipment to carry out, which limits their potential use in many resource-poor settings (19). Therefore, successful application of these test faces many challenges in the pathway from discovery to final use.

Some studies have shown that poor immune status was one characteristic of ATB patients, suggesting that the evaluation of host immunity could be applied as a potential direction for TB diagnosis and monitoring (13, 20–22). Unfortunately, difficulties existed in host immunity evaluation of ATB patients due to the limitations of current tests such as lymphocyte subset analysis and the measurement of serum protein (23). These available methods could not fully reflect the immune status of host. Our team have previously developed lymphocyte non-specific function detection-a novel approach for evaluating host immunity based on phorbol-12-myristate-13-acetate (PMA)/ionomycin stimulation. And we have confirmed the performance of this method for host immunity evaluation in a variety of diseases including infection and autoimmune diseases (24, 25). Thus, it is worth considering whether lymphocyte non-specific function detection could be applied to diagnose TB. On the other hand, TB-specific antigen (TBAg)/phytohemagglutinin (PHA) ratio has been proposed as a potential diagnostic candidate for ATB by Wang and his colleague in recent years (26, 27). This simple calculation made it possible for T-SPOT to distinguish between ATB and LTBI by dividing the spot number of TBAg well by that of PHA well (28). However, the spot number of PHA well might be inaccurate in case of high value (29). Although our previous study showed that reducing the number of cells added to the PHA well could improve the accuracy of the results, this improvement requires an additional operation and might not be suitable for clinical application (30). Besides, TBAg/PHA ratio was helpless in identifying ATB with negative T-SPOT results due to its computational limitations. Several studies have demonstrated that the combination of multiple indicators could promote ATB diagnosis (31, 32). Accordingly, we speculate that the use of the combination of lymphocyte non-specific function (non-specific marker) and T-SPOT (TB-specific marker) has the potential to improve rapid differential diagnosis between ATB and LTBI. Consequently, we investigated the potential value of lymphocyte non-specific function detection and its combination with T-SPOT for determining MTB infection status by enrolling subjects with ATB and LTBI. We demonstrated the advantages of utilizing lymphocyte non-specific function detection for the analysis of patients with MTB infection.

## Methods

### Study Design

Adult participants aged 18 years or older were enrolled for performing T-SPOT assay and lymphocyte non-specific function detection at Tongji hospital from January 2019 to October 2020. Patients with ATB, individuals with LTBI, and healthy controls (HC) were identified and recruited based on laboratory and clinical evaluation. ATB was diagnosed by positive GeneXpert MTB/RIF or mycobacterial culture on sputum or bronchoalveolar lavage fluid with symptoms compatible of ATB including prolonged cough, chest pain, weakness or fatigue, weight loss, fever, and night sweats. Participants receiving anti-TB medication in two months prior to the enrollment were excluded from the analysis. LTBI was defined by a positive T-SPOT test without symptomatic, microbiological, or radiological evidences of ATB and history of TB. HC was defined by a negative T-SPOT test, while without any symptoms or signs of diseases. The laboratory scientists who performed the immunological and microbiological assays were blinded to the clinical data including disease status of participants. This study was reviewed and approved by the committee of Tongji hospital, Tongji Medical College, Huazhong University of Science and Technology.

### Lymphocyte Non-Specific Function Assay

PMA/ionomycin-stimulated lymphocyte non-specific function assay was performed as described previously (24). The procedures are described in brief as following: (1) 100 µl of heparinized venous blood was diluted with 400 µl of IMDM medium; (2) the diluted sample was incubated in the presence of Leukocyte Activation Cocktail (Becton Dickinson GolgiPlug™) for 4 h; (3) the cells were labeled with antibodies (anti-CD45, anti-CD3, anti-CD4, anti-CD8, and anti-CD56) (BD Biosciences); (4) the cell were fixed and permeabilized; (5) the cells were stained with intracellular anti-interferon-gamma (IFN-γ) antibody (BD Biosciences); and (6) the cells were analyzed with FACSCanto flow cytometer. The percentages of IFN-γ^+^ cells in different cell subsets were defined as the non-specific function of them (**Supplementary Figure 1**) (e.g., the percentage of IFN-γ^+^ cells in CD3^+^CD4^+^CD8^-^ cells was regarded as the non-specific function of CD4^+^ T cells; the percentage of IFN-γ^+^ cells in CD3^+^CD4^-^CD8^+^ cells was regarded as the non-specific function of CD8^+^ T cells; the percentage of IFN-γ^+^ cells in CD3^-^CD56^+^ cells was regarded as the non-specific function of NK cells). Given that the background is very low in the assay (the proportion of IFN-γ^+^ cells under 0.1%), we did not subtract the background when reporting lymphocyte non-specific function.

### T-SPOT Assay

Heparin anticoagulated peripheral blood was collected and analyzed using T-SPOT assay (Oxford Immunotec, Oxford, UK) according to the manufacturer’s instructions. Briefly, peripheral blood mononuclear cells (PBMCs) were separated by Ficoll−Hypaque gradient centrifugation. Then, the isolated PBMCs (2.5 × 10^5^) were added to 96-well plates precoated with antibody against IFN−γ. Four wells were used for each subject: medium well, early secreted antigenic target 6 (ESAT-6) well, culture filtrate protein 10 (CFP-10) well, and PHA well. Plates were incubated for 16-20 h at 37 C° with 5% CO_2_ and developed using an anti-IFN-γ antibody conjugate and substrate to detect the presence of secreted IFN-γ. Spot-forming cells (SFC) were counted with an automated enzyme−linked immunospot (ELISPOT) reader (CTL Analyzers, Cleveland, OH, USA). The test result was positive if ESAT-6 and/or CFP-10 spot number minus negative control spot number ≥ 6. The test result was negative if both ESAT-6 spot number minus negative control spot number and CFP-10 spot number minus negative control spot number ≤ 5. Results were considered undetermined if the spot number in the PHA well were < 20 or if spot number in the medium well were > 10. The ratio of ESAT-6 SFC to PHA SFC (ESAT-6/PHA ratio) and CFP-10 SFC to PHA SFC (CFP-10/PHA ratio) were calculated. The larger of the above two values was defined as the TBAg/PHA ratio of one participant.

### Statistical Analysis

Continuous variables were expressed as means ± standards deviation (SD) or median (interquartile range). Categorical variables were expressed as number (%). Comparison was performed using Mann-Whitney *U* test for continuous variables and Chi-square test or Fisher’s exact test for categorical variables. All statistical tests were two sided. Statistical significance was considered when *P* < 0.05. For the identification of a diagnostic model, all variables with statistical significance were taken as candidates for multivariable logistic regression analyses, and the regression equation (diagnostic model) was obtained. The regression coefficients of the model were regarded as the weights for the respective variables, and a score for each participant was calculated. The performance of various indicators was evaluated by the receiver operating characteristic (ROC) curve analysis. Area under the curve (AUC), sensitivity, specificity, positive predictive value (PPV), negative predictive value (NPV), positive likelihood ratio (PLR), negative likelihood ratio (NLR), and accuracy, together with their 95% confidence intervals (CI), were calculated. The AUCs were compared using the z statistic with the procedure of Delong et al. (33). Data were analyzed using SPSS version 25.0 (SPSS, Inc., Chicago, IL, USA), MedCalc version 11.6 (MedCalc, Mariakerke, Belgium), GraphPad Prism version 8 (GraphPad Software, San Diego, CA, USA), and R 4.0.2 program (R Core Team).

## Results

### Participants’ Characteristics

A total of 208 participants including 49 ATB patients, 64 LTBI individuals, and 95 HC were consecutively enrolled to the analysis. All included participants were HIV-negative. No difference was observed between ATB and LTBI in respect to the distribution of age and sex (**Table 1**). Among 49 patients diagnosed as ATB, 10 subjects had negative T-SPOT results, while the remaining 39 had positive T-SPOT results. **Table 2** described the characteristics of ATB subjects with negative T-SPOT results and those with positive T-SPOT results. There was no evidence of difference in the distribution of genders and underling diseases between the groups.

**Table 1:** Demographic and clinical characteristics of included subjects.

Variables	ATB (n = 49)	LTBI (n = 64)	HC (n = 95)
Age, years	48 (29-60)	50 (40-61)	47 (33-57)
Sex, male, %	27 (55.1%)	41 (64.06%)	60 (63.16%)
TB history	11 (22.45%)	0 (0%)	0 (0%)
Underlying condition or illness			
Diabetes mellitus	7 (14.29%)	8 (12.5%)	0 (0%)
Solid tumor	5 (10.2%)	6 (9.38%)	0 (0%)
Hematological malignancy	1 (2.04%)	1 (1.56%)	0 (0%)
End-stage renal disease	4 (8.16%)	7 (10.94%)	0 (0%)
Liver cirrhosis	2 (4.08%)	3 (4.69%)	0 (0%)
Organ transplantation	2 (4.08%)	1 (1.56%)	0 (0%)
Immunosuppressive condition^*^	7 (14.29%)	4 (6.25%)	0 (0%)
Positive mycobacterial culture	40 (81.63%)	NA	NA
Positive GeneXpert MTB/RIF	36 (73.47%)	NA	NA

ATB, active tuberculosis; LTBI, latent tuberculosis infection; HC, healthy controls; TB, tuberculosis; NA, not applicable. ^*^Patients who underwent organ transplantation, chemotherapy or took immunosuppressants within 3 months. Data were presented as medians (25th-75th percentiles) or numbers (percentages).

**Table 2:** Demographic and clinical characteristics of ATB patients with negative and positive T-SPOT results.

Variables	ATB with negative T-SPOT result (n = 10)	ATB with positive T-SPOT result (n = 39)	P*
Age, years	52 (26-56)	48 (29-61)	0.673
Sex, male, %	5 (50%)	22 (56.41%)	0.716
TB history	3 (30%)	8 (20.51%)	0.521
Presence of BCG scar	4 (40%)	17 (43.59%)	0.838
Underlying condition or illness			
Diabetes mellitus	2 (20%)	5 (12.82%)	0.563
Solid tumor	1 (10%)	4 (10.26%)	0.981
Hematological malignancy	0 (0%)	1 (2.56%)	1
End-stage renal disease	1 (10%)	3 (7.69%)	1
Liver cirrhosis	0 (0%)	2 (5.13%)	1
Organ transplantation	1 (10%)	1 (2.56%)	0.37
Immunosuppressive condition^†^	2 (20%)	5 (12.82%)	0.563
Positive mycobacterial culture	8 (80%)	32 (82.05%)	0.881
Positive GeneXpert MTB/RIF	7 (70%)	29 (74.36%)	0.781

ATB, active tuberculosis; BCG, bacille Calmette-Guérin; TB, tuberculosis *Comparisons were performed between these two groups using Mann-Whitney U test, chi-square test, or Fisher’s exact test. ^†^Patients who underwent organ transplantation, chemotherapy, or took immunosuppressants within 3 months. Data were presented as medians (25th-75th percentiles), or numbers (percentages).

### The Correlation Between PHA Spot Number and Lymphocyte Non-Specific Function

**Figure 1:**
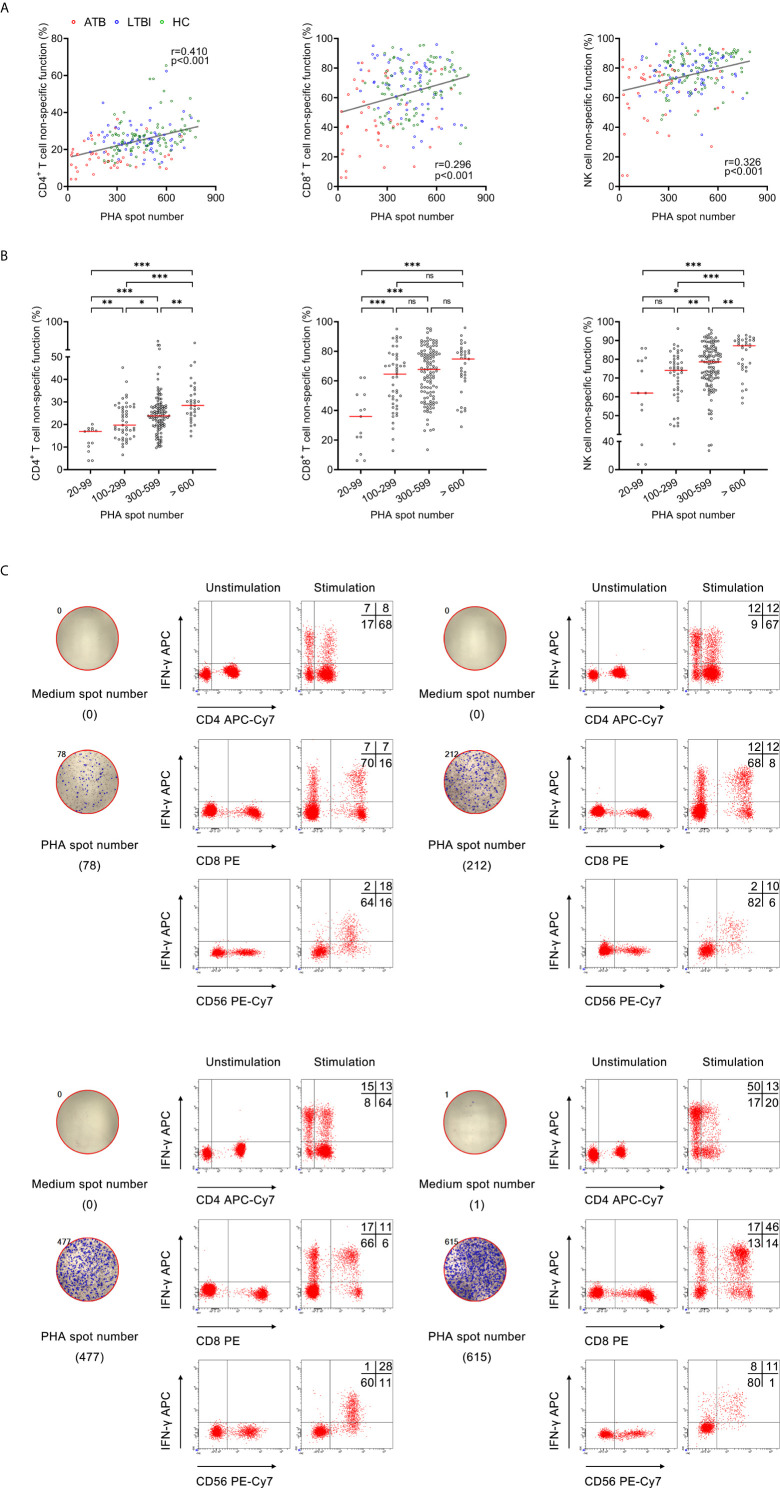
The relationship between PHA spot number and lymphocyte non-specific function. **(A)** Correlation between PHA spot number and non-specific function of CD4^+^ T cells, CD8^+^ T cells, and NK cells in 208 participants. Each symbol represents an individual donor. **(B)** Scatter plots showing the results of CD4^+^ T cell non-specific function, CD8^+^ T cell non-specific function, and NK cell non-specific function under different PHA spot number. Horizontal lines indicate the median. *P < 0.05; **P < 0.01; ***P < 0.001; ns, no significance (Mann-Whitney *U* test). **(C)** Flow plots showing the representative results of CD4^+^ T cell non-specific function, CD8^+^ T cell non-specific function, and NK cell non-specific function under different PHA spot number. PHA, phytohemagglutinin.

We examined the correlation between PHA spot number and lymphocyte non-specific function. It was observed that significantly positive correlation existed between PHA spot number and CD4^+^ T cell non-specific function (r=0.410, *P*<0.001), CD8^+^ T cell non-specific function (r=0.296, *P*<0.001), and NK cell non-specific function (r=0.326, *P*<0.001) (**Figure 1A**). Furthermore, we stratified PHA spot number and found the trend that lymphocyte non-specific function increased with the increasing PHA spot number (**Figures 1B, C** **)**.

### Lymphocyte Non-Specific Function for Identifying ATB With False-Negative T-SPOT Result

**Figure 2:**
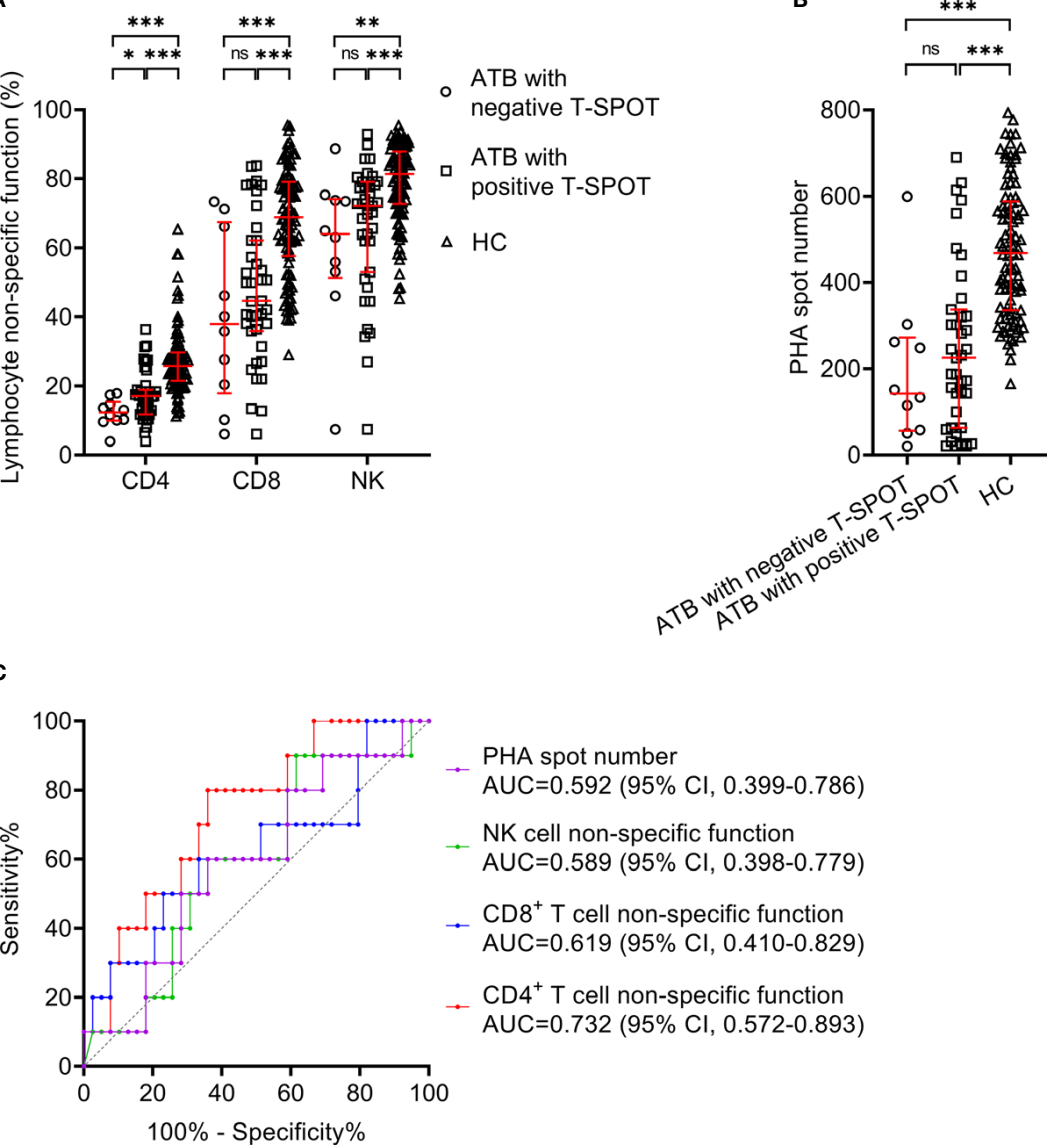
Lymphocyte non-specific function detection for identifying ATB patients with false-negative T-SPOT result. **(A)** Scatter plots showing the results of lymphocyte non-specific function in ATB with negative T-SPOT result (n=10),ATB with positive T-SPOT result (n=39) and HC (n=95). Bars indicated the medians and interquartile ranges. *P < 0.05; **P < 0.01; ***P < 0.001; ns, no significance (Mann-Whitney *U* test). **(B)** Scatter plots showing PHA spot number in ATB with negative T-SPOT result (n=10), ATB with positive T-SPOT result (n=39) and HC (n=95). Bars indicated the medians and interquartile ranges. ***P < 0.001; ns, no significance (Mann-Whitney *U* test). **(C)** ROC analysis showing the performance of CD4^+^ T cell non-specific function, CD8^+^ T cell non-specific function, NK cell non-specific function, and PHA spot number in distinguishing ATB patients with negative T-SPOT result from those with positive T-SPOT result. ATB, active tuberculosis; HC, healthy controls; PHA, phytohemagglutinin; ROC, receiver operating characteristic; AUC, area under the curve; CI, confidence interval.

We compared lymphocyte non-specific function between ATB with negative T-SPOT result and those with positive T-SPOT result. It was found that CD4^+^ T cell non-specific function was significantly lower in patients with negative T-SPOT result than in those with positive T-SPOT results, while no significant difference presented in CD8^+^ T cell non-specific function and NK cell non-specific function between these two groups (**Figure 2A**). When comparing with HC, the lymphocyte non-specific function of ATB was significantly lower regardless of T-SPOT results (**Figure 2A**). Besides, there was no significant difference between the two groups in PHA spot number (**Figure 2B**). Further ROC curve analysis showed that CD4^+^ T cell non-specific function had an AUC of 0.732 (95% CI, 0.572-0.893) for discriminating negative T-SPOT results from positive T-SPOT results among ATB patients (**Figure 2C**).

### Lymphocyte Non-Specific Function for Distinguishing Between ATB and LTBI

**Figure 3:**
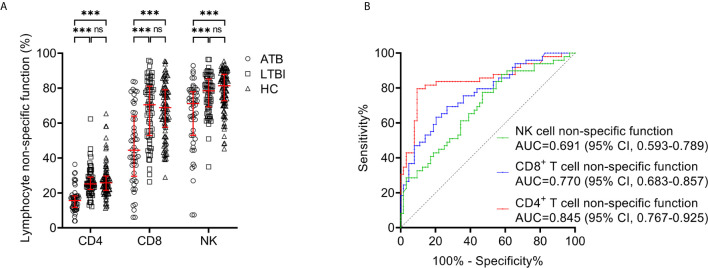
Lymphocyte non-specific function detection for distinguishing between ATB and LTBI. **(A)** Scatter plots showing the results of CD4^+^ T cell non-specific function, CD8^+^ T cell non-specific function, and NK cell non-specific function in ATB patients (n=49), LTBI individuals (n=64), and HC (n=95). Bars indicated the medians and interquartile ranges. ***P < 0.001; ns, no significance (Mann-Whitney *U* test). **(B)** ROC analysis showing the performance of CD4^+^ T cell non-specific function, CD8^+^ T cell non-specific function, and NK cell non-specific function in discriminating ATB patients from LTBI individuals. ATB, active tuberculosis; LTBI, latent tuberculosis infection; HC, healthy controls; ROC, receiver operating characteristic; AUC, area under the curve; CI, confidence interval.

We compared lymphocyte non-specific function between ATB patients and LTBI individuals. It was found that the non-specific function of CD4^+^ T cells, CD8^+^ T cells, and NK cells was significantly lower in patients diagnosed with ATB than those with LTBI (**Figure 3A**). While no significant difference was observed in lymphocyte non-specific function between LTBI and HC (**Figure 3A**). And then we performed ROC curve analysis. The results of the diagnostic accuracy of lymphocyte non-specific function in distinguishing ATB from LTBI were shown in **Figure 3B**. The AUCs presented by CD4^+^ T cell non-specific function, CD8^+^ T cell non-specific function, and NK cell non-specific function were 0.845 (95% CI, 0.767-0.925), 0.770 (95% CI, 0.683-0.857), 0.691 (95% CI, 0.593-0.789) respectively (**Figure 3B**, **Table 3**). Concretely, when 11.7% was used as the threshold, the sensitivity and specificity of CD4^+^ T cell non-specific function for distinguishing ATB from LTBI was 61.22% (95% CI, 47.25%-73.57%) and 90.63% (95% CI, 81.02%-95.63%), respectively. The cutoff value of 41.6% for CD8^+^ T cell non-specific function showed a sensitivity of 46.94% (95% CI, 33.70%-60.62%) and specificity of 90.63% (95% CI, 81.02%-95.63%). The sensitivity and specificity for NK cell non-specific function were 28.57% (95% CI, 17.85%-42.41%) and 90.63% (95% CI, 81.02%-95.63%) with the threshold as 61.7% (**Table 3**).

**Table 3:** The performance of various indicators for distinguishing between ATB and LTBI.

Variables	Cutoff value	AUC (95% CI)	Sensitivity (95% CI)	Specificity (95% CI)	PPV (95% CI)	NPV (95% CI)	PLR (95% CI)	NLR (95% CI)	Accuracy
CFP-10 spot number	34	0.649 (0.540-0.758)	36.73% (24.66%-50.73%)	90.63% (81.02%-95.63%)	75.00% (55.10%-88.00%)	65.17% (54.83%-74.25%)	3.92 (1.68-9.13)	0.70 (0.56-0.88)	67.26%
CD4^+^ T cell non-specific function (%)	17.3	0.845 (0.767-0.925)	61.22% (47.25%-73.57%)	90.63% (81.02%-95.63%)	83.33% (68.11%-92.13%)	75.32% (64.65%-83.60%)	6.53 (2.95-14.44)	0.43 (0.30-0.61)	77.88%
CD8^+^ T cell non-specific function (%)	41.6	0.770 (0.683-0.857)	46.94% (33.70%-60.62%)	90.63% (81.02%-95.63%)	79.31% (61.61%-90.16%)	69.05% (58.51%-77.92%)	5.01 (2.21-11.34)	0.59 (0.44-0.77)	71.68%
NK cell non-specific function (%)	61.7	0.691 (0.593-0.789)	28.57% (17.85%-42.41%)	90.63% (81.02%-95.63%)	70.00% (48.10%-85.45%)	62.37% (52.21%-71.54%)	3.05 (1.26-7.36)	0.79 (0.65-0.96)	63.72%
Diagnostic model	0.57	0.939 (0.898-0.981)	83.67% (70.96%-91.49%)	90.63% (81.02%-95.63%)	87.23% (74.83%-94.02%)	87.88% (77.86%-93.73%)	8.93 (4.13-19.31)	0.18 (0.10-0.34)	87.61%

ATB, active tuberculosis; LTBI, latent tuberculosis infection; CFP-10, culture filtrate protein 10; AUC, area under the curve; PPV, positive predictive value; NPV, negative predictive value; PLR, positive likelihood ratio; NLR, negative likelihood ratio; CI, confidence interval.

### Combination of Lymphocyte Non-Specific Function and T-SPOT for Differentiating ATB From LTBI

**Figure 4:**
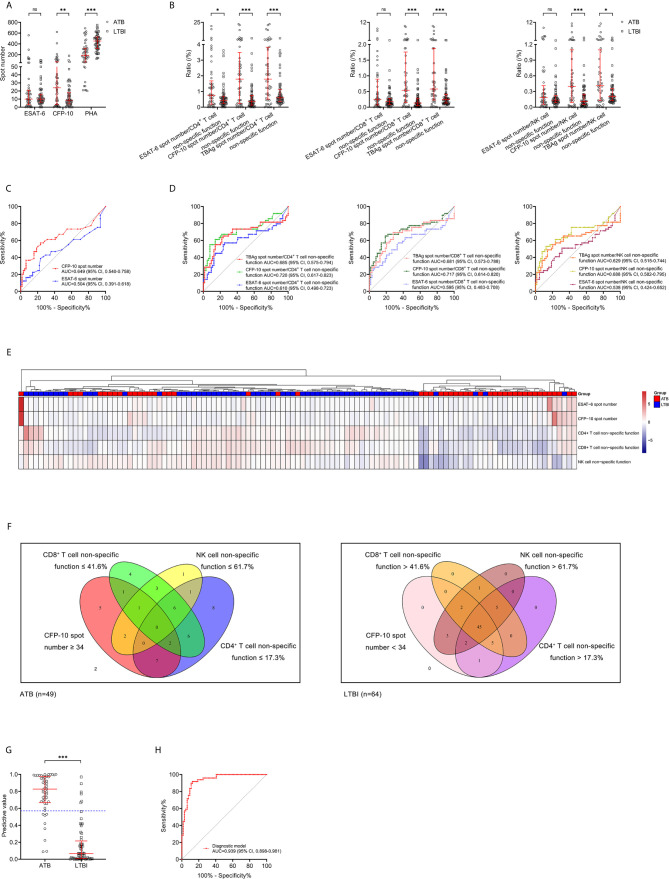
Combination of lymphocyte non-specific function and T-SPOT for differentiating ATB from LTBI. **(A)** Scatter plots showing the results of ESAT-6 spot number, CFP-10 spot number, and PHA spot number in ATB patients (n=49) and LTBI individuals (n=64). Bars indicated the medians and interquartile ranges. **P < 0.01; ***P < 0.001; ns, no significance (Mann-Whitney *U* test). **(B)** Scatter plots showing the ratio of MTB-specific antigen spot number/lymphocyte non-specific function in ATB patients (n=49) and LTBI individuals (n=64). Bars indicated the medians and interquartile ranges. *P < 0.05; ***P < 0.001; ns, no significance (Mann-Whitney *U* test). **(C)** ROC analysis showing the performance of ESAT-6 spot number and CFP-10 spot number in discriminating ATB patients from LTBI individuals. **(D)** ROC analysis showing the performance of MTB-specific antigen spot number/lymphocyte non-specific function in discriminating ATB patients from LTBI individuals. **(E)** Heatmap showing the cluster analysis of lymphocyte non-specific function and T-SPOT results in ATB patients (n=49) and LTBI individuals (n=64). Each rectangle indicates a result of a subject. **(F)** Venn diagrams showing the overlap of CD4^+^ T cell non-specific function, CD8^+^ T cell non-specific function, NK cell non-specific function, and CFP-10 spot number in ATB patients (n=49) and LTBI individuals (n=64). **(G)** Scatter plots showing the predictive value of diagnostic model in ATB patients (n=49) and LTBI individuals (n=64). Bars indicated the medians and interquartile ranges. ***P<0.001 (Mann-Whitney *U* test). Blue dotted line indicates the cutoff value in distinguishing these two groups. **(H)** ROC analysis showing the performance of diagnostic model based on the combination of CD4+ T cell non-specific function, NK cell non-specific function, and CFP-10 spot number in discriminating ATB patients from LTBI individuals. ESAT-6, early secreted antigenic target 6; CFP-10, culture filtrate protein 10; PHA, phytohemagglutinin; MTB, *Mycobacterium tuberculosis*; ATB, active tuberculosis; LTBI, latent tuberculosis infection; ROC, receiver operating characteristic; AUC, area under the curve; CI, confidence interval.

The comparison in T-SPOT between ATB and LTBI was performed. No difference was observed between ATB and LTBI in ESAT-6 spot number while CFP-10 spot number was significantly higher in ATB patients than that in LTBI individuals (**Figure 4A**). ROC curve analysis indicated limited performance for ESAT-6 spot number and CFP-10 spot number (**Figure 4C**). Given that the pattern of lymphocyte non-specific function was inverse to that observed by T-SPOT between ATB and LTBI, we predicted that the combination of these two assays would be leveraged for improving the stratification of patient groups. Consequently, we calculated the ratio of ESAT-6, CFP-10, or TBAg spot number to lymphocyte non-specific function. We found the ratios were obviously higher in ATB patients than LTBI individuals (**Figure 4B**). However, the discriminatory power measured by the AUC presented lower than 0.75 for all ratios (**Figure 4D**). Nonetheless, further cluster analysis using heatmap and the overlap between lymphocyte non-specific function and T-SPOT assay still showed that the combination of these two assays might improve the diagnostic value (**Figures 4E, F**). To establish the diagnostic model based on a combination of the two approaches for distinguishing ATB from LTBI, all variables with statistical significance were used for multivariable logistic regression analysis. The diagnostic model in distinguishing ATB from LTBI were built as follows: P = 1/[1 + e^-(0.039*CFP-10 spot number - 0.363*CD4+ T cell non-specific function - 0.052*NK cell non-specific function + 9.679)^] P, predictive value; e, natural logarithm. The three-marker diagnostic model distinguished patients with ATB disease from those with LTBI with an AUC of 0.939 (95% CI, 0.898-0.981) and demonstrated a sensitivity and specificity of 83.67% (95% CI, 70.96%-91.49%) and 90.63% (95% CI, 81.02%-95.63%) respectively while using 0.57 as the threshold (**Table 3**, **Figures 4G, H** **)**. Z tests between the ROC curves showed a significant improvement for the model compared to either lymphocyte non-specific function (CD4^+^ T cell non-specific function, *P*=0.011; CD8^+^ T cell non-specific function, *P*<0.001; NK cell non-specific function, *P*<0.001) or T-SPOT (ESAT-6 spot number, *P*<0.001; CFP-10 spot number, *P*<0.001).

### Comparison Between Diagnostic Model and TBAg/PHA Ratio for Discriminating ATB From LTBI

**Figure 5:**
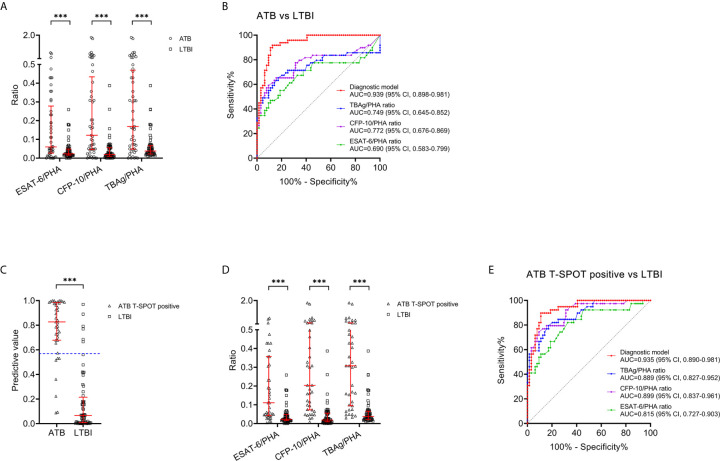
Comparison between the diagnostic model and TBAg/PHA ratio for discriminating ATB from LTBI. **(A)** Scatter plots showing the values of ESAT-6/PHA ratio, CFP-10/PHA ratio, and TBAg/PHA ratio in ATB patients (n=49) and LTBI individuals (n=64). Bars indicated the medians and interquartile ranges. ***P < 0.001 (Mann-Whitney *U* test). **(B)** ROC analysis showing the performance of ESAT-6/PHA ratio, CFP-10/PHA ratio, TBAg/PHA ratio, and the diagnostic model in discriminating ATB patients from LTBI individuals. **(C)** Scatter plots showing the predictive value of diagnostic model in ATB patients with positive T-SPOT result (n=39) and LTBI individuals (n=64). Bars indicated the medians and interquartile ranges. ***P < 0.001 (Mann-Whitney *U* test). **(D)** Scatter plots showing the values of ESAT-6/PHA ratio, CFP-10/PHA ratio, and TBAg/PHA ratio in ATB patients with positive T-SPOT result (n=39) and LTBI individuals (n=64). Bars indicated the medians and interquartile ranges. ***P < 0.001 (Mann-Whitney *U* test). **(E)** ROC analysis showing the performance of ESAT-6/PHA ratio, CFP-10/PHA ratio, TBAg/PHA ratio, and the diagnostic model in discriminating ATB patients with positive T-SPOT result from LTBI individuals. ESAT-6, early secreted antigenic target 6; CFP-10, culture filtrate protein 10; PHA, phytohemagglutinin; TBAg, tuberculosis-specific antigen; ATB, active tuberculosis; LTBI, latent tuberculosis infection; ROC, receiver operating characteristic; AUC, area under the curve; CI, confidence interval.

The value of TBAg/PHA ratio for distinguishing between ATB patients and LTBI individuals was also analyzed. ESAT-6/PHA ratio, CFP-10/PHA ratio, and TBAg/PHA ratio were found significantly higher in patients with ATB than individuals with LTBI (**Figure 5A**). Further ROC curve analysis showed that ESAT-6/PHA ratio, CFP-10/PHA ratio, and TBAg/PHA ratio had AUCs of 0.690 (95% CI, 0.583-0.799), 0.772 (95% CI, 0.676-0.869), and 0.749 (95% CI, 0.645-0.852) for discriminating ATB from LTBI, in comparison to 0.939 (95% CI, 0.898-0.981) for the diagnostic model (**Table 4**, **Figure 5B**). The AUC of diagnostic model was superior than those achieved for the individual ratios in T-SPOT (ESAT-6/PHA ratio, *P*<0.001; CFP-10/PHA ratio, *P*=0.001; TBAg/PHA ratio, *P*<0.001). In addition, we evaluated the utility of these indicators for discriminating ATB from LTBI among subjects with positive T-SPOT results. The diagnostic model presented an AUC of 0.935 (95% CI, 0.890-0.981), which was comparable to that obtained based on all patients previously (**Figures 5C, E** **)**. While the performance of ESAT-6/PHA ratio, CFP-10/PHA ratio, and TBAg/PHA ratio was obviously increased. The AUCs of ESAT-6/PHA ratio, CFP-10/PHA ratio, and TBAg/PHA ratio were 0.815 (95% CI, 0.727-0.903), 0.899 (95% CI, 0.837-0.961), and 0.889 (95% CI, 0.827-0.952), respectively (**Figures 5D, E** **)**. However, our established diagnostic model still showed superior or comparable performance compared to the ratios (ESAT-6/PHA ratio, *P*=0.017; CFP-10/PHA ratio, *P*=0.298; TBAg/PHA ratio *P*=0.219) (**Table 5**).

**Table 4:** The value of ESAT-6/PHA ratio, CFP-10/PHA ratio and TBAg/PHA ratio for discriminating ATB from LTBI.

Variables	Cutoff value	AUC (95% CI)	Sensitivity (95% CI)	Specificity (95% CI)	PPV (95% CI)	NPV (95% CI)	PLR (95% CI)	NLR (95% CI)	Accuracy
ESAT-6/PHA ratio	0.13	0.691 (0.583-0.799)	40.82% (28.21%-54.75%)	92.19% (82.98%-96.62%)	80.00% (60.87%-91.14%)	67.05% (56.69%-75.97%)	5.22 (2.11-12.94)	0.64 (0.50-0.82)	69.91%
CFP-10/PHA ratio	0.115	0.772 (0.676-0.869)	53.06% (39.38%-66.30%)	93.75% (85.00%-97.54%)	86.67% (70.32%-94.69%)	72.29% (61.84%-80.77%)	8.49 (3.17-22.73)	0.50 (0.37-0.68)	76.11%
TBAg/PHA ratio	0.16	0.749 (0.645-0.852)	53.06% (39.38%-66.30%)	90.63% (81.02%-95.63%)	81.25% (64.69%-91.11%)	71.60% (60.98%-80.27%)	5.66 (2.53-12.67)	0.52 (0.38-0.70)	74.34%

ESAT-6, early secreted antigenic target 6; CFP-10, culture filtrate protein 10; ATB, active tuberculosis; LTBI, latent tuberculosis infection; AUC, area under the curve; PPV, positive predictive value; NPV, negative predictive value; PLR, positive likelihood ratio; NLR, negative likelihood ratio; CI, confidence interval.

**Table 5:** The performance of various methods for differentiating ATB from LTBI in participants with positive T-SPOT results.

Variables	Cutoff value	AUC (95% CI)	Sensitivity (95% CI)	Specificity (95% CI)	PPV (95% CI)	NPV (95% CI)	PLR (95% CI)	NLR (95% CI)	Accuracy
ESAT-6/PHA ratio	0.13	0.815 (0.727-0.903)	48.72% (33.86%-63.80%)	92.19% (82.98%-96.62%)	79.17% (59.53%-90.76%)	74.68% (64.11%-82.97%)	6.24 (2.53-15.35)	0.56 (0.41-0.76)	75.73%
CFP-10/PHA ratio	0.115	0.899 (0.837-0.961)	66.67% (50.98%-79.37%)	93.75% (85.00%-97.54%)	86.67% (70.32%-94.69%)	82.19% (71.88%-89.29%)	10.67 (4.03-28.26)	0.36 (0.23-0.56)	83.50%
TBAg/PHA ratio	0.16	0.889 (0.827-0.952)	64.10% (48.42%-77.26%)	90.63% (81.02%-95.63%)	80.65% (63.72%-90.81%)	80.56% (69.97%-88.05%)	6.84 (3.08-15.17)	0.40 (0.26-0.61)	80.58%
Diagnostic model	0.57	0.936 (0.890-0.981)	82.05% (67.33%-91.02%)	90.63% (81.02%-95.63%)	84.21% (69.58%-92.56%)	89.23% (79.40%-94.69%)	8.75 (4.03-19.01)	0.20 (0.10-0.39)	87.38%

ATB, active tuberculosis; LTBI, latent tuberculosis infection; ESAT-6, early secreted antigenic target 6; CFP-10, culture filtrate protein 10; AUC, area under the curve; PPV, positive predictive value; NPV, negative predictive value; PLR, positive likelihood ratio; NLR, negative likelihood ratio; CI, confidence interval.

## Discussion

The TB continues relentlessly, especially during the current global COVID-19 pandemic, killing more than other infection, while the progress being lagging behind other major infectious diseases (34–38). A fundamental issue with controlling the disease is the inadequacy of current available tests for stratification of status of MTB infection (39). Multiple disadvantages present including unsatisfactory sensitivity, high cost as well as reliance on complicated infrastructure. Novel approaches are needed to overcome the limitations of existing immunodiagnostic tests, including their inability for differentiating between ATB and LTBI.

There was rare study elucidating lymphocyte non-specific function detection for stratifying MTB infection. Our study benchmarked the value of lymphocyte non-specific function in the diagnosis of ATB for the first time. It was observed that CD4^+^ T cell non-specific function showed certain potential in identifying T-SPOT-negative ATB patients. Furthermore, we found that lymphocyte non-specific function detection including CD4^+^ T cell non-specific function, CD8^+^ T cell non-specific function, and NK cell non-specific function could be used to distinguish between ATB and LTBI. In view of the fact that the combination of multiple parameters may perform better than single parameter alone in many studies (20, 21, 40), we further successfully established a three-index diagnostic model that could discriminate ATB from LTBI with good utility based on the combination of lymphocyte non-specific function and T-SPOT assay. The diagnostic model based on the combination of CD4^+^ T cell non-specific function, NK cell non-specific function, and CFP-10 spot number established in the present study was 90.63% specific and identified 83.67% of ATB cases, indicating that it could be used as a rule-in ATB diagnostic test to allow rapid treatment initiation. Furthermore, we compared our established model with TBAg/PHA ratio-another TB diagnostic indicator reported in the previous studies and found that the diagnostic performance of our model was better than that of TBAg/PHA ratio.

Confirming previous reports, T-SPOT based on detection an immune response under TB-specific antigen stimulation could not stratify ATB and LTBI well (13, 41, 42). Only CFP-10 spot number showed certain potential for differential diagnosis of ATB and LTBI in the current study. We first analyzed the utility of the ratio of TB-specific antigen spot number to lymphocyte non-specific function in distinguishing ATB from LTBI, but unfortunately, no great effect was observed. However, we found the potential of the combination of lymphocyte non-specific function and T-SPOT in determining the status of TB infection through the analysis of Venn diagram and heatmap and we further successfully established a diagnostic model through multivariable logistic regression. TBAg/PHA ratio is a simple calculation based on T-SPOT assay itself. We compared its performance with the model we established in distinguishing ATB from LTBI and found that our model was significantly better than TBAg/PHA ratio. Notably, we observed that most of the previous studies targeted for the diagnostic performance of TBAg/PHA ratio were based on T-SPOT-positive subjects. Thus, the inclusion of T-SPOT-negative patients in our study would reduce the value of TBAg/PHA ratio. Therefore, we additionally compared the ability of two methods to differentiate ATB from LTBI in T-SPOT-positive patients. It was observed that the performance of the diagnostic model hardly changed while the performance of TBAg/PHA ratio was obviously increased. But the AUCs of CFP-10/PHA ratio and TBAg/PHA ratio were still lower than that of diagnostic model. These findings indicated the robustness and superiority of our established model.

An interesting question is why the combination of TBAg spot number and lymphocyte non-specific function is better than TBAg/PHA ratio in distinguishing ATB from LTBI. We think there are two reasons to explain this issue. First, lymphocyte non-specific function detection is better and more comprehensive than the number of PHA spots in reflecting host immunity. Although we found a significantly positive correlation between lymphocyte non-specific function and the number of PHA spots, the number of PHA spots signifies the broad-spectrum response of lymphocytes to PHA. In addition, the r values observed in the correlation between lymphocyte non-specific function and PHA spot number also reflected high variability among different patients. While lymphocyte non-specific function detection could reflect the ability of activation, chemotaxis, and cytotoxicity of lymphocytes (43). Second, lymphocyte non-specific function detection is more stable than the readout of PHA spot number. The counting of PHA spot number would have poor repeatability due to the experimental operation and inaccuracy in reading high values (29). On the contrary, our previous results showed that lymphocyte non-specific function detection was extremely stable (coefficients of variations within 5%) (24). Therefore, lymphocyte non-specific function is superior to PHA spot number, especially as a reproducible and widely accepted diagnostic indicator. Another question is why ESAT-6 spot number was not included to the diagnostic model. We think this may due to the significantly positive correlation between ESAT-6 spot number and CFP-10 spot number (**Supplementary Figure 2**). However, one kind of situation often occurs, that is, some patients only show ESAT-6 response. But our diagnostic model only included CFP-10 spot number. Therefore, we also established a model incorporating ESAT-6 spot number. We found the AUC produced by this model was similar to the former (**Supplementary Figure 3**).

Negative T-SPOT result in ATB was a common phenomenon (44, 45). Although some studies have explored the reasons for these negative results, this issue has not been fully explained. The occurrence of false-negative T-SPOT results in microbiologically confirmed ATB was generally considered to be partially caused by some host factors such as immunosuppression or malnutrition (44, 46). Nevertheless, due to the lack of uniform and reliable assessment methods for host immunity, most studies embodied this concept with the patient’s underlying diseases, age, or the number of lymphocytes (45–47). In this study, we introduced lymphocyte non-specific function to this field for the first time and discovered the potential of lymphocyte non-specific function represented by CD4^+^ T cell. It was undeniable that its value is limited with an AUC of only 0.72. But this may also indicate that the appearance of false-negative T-SPOT results is more likely to be caused by a variety of factors, such as the breadth of antigen coverage and defective cell response. Nonetheless, it might improve diagnostic accuracy when used in conjunction with other indicators. Further research targeted for multi-dimensional explanation need to be carried out.

Several limitations should be noticed in this study. First, given the relatively small sample size is an obvious caveat of our study, an independent cohort with larger population should be replicated in the future. Second, since both T-SPOT and flow cytometry were unusually used in TB-endemic setting with low income, the diagnostic model established in the present study might be not applicable in these areas. Nevertheless, our model could help validate other low-cost methods. Third, regarding that infection including HIV infection and COVID-19 might affect the IFN-γ secretion of lymphocyte, further investigation targeted for the influence of underlying conditions such as co-infection on lymphocyte non-specific function detection are needed in the future. Fourth, due to the requirement for elaborate procedures, expensive equipment, and well-trained personnel, the applicability of the model might be limited in clinical practice. Finally, since we did not dynamically monitor lymphocyte non-specific function during anti-TB treatment in the present study, further well-designed studies should be conducted to clarify the benefit and efficacy from lymphocyte non-specific function based immune monitoring.

In summary, our findings demonstrated the ability to classify ATB patients and LTBI individuals by detecting lymphocyte non-specific function and the potential advantages of combining lymphocyte non-specific function detection and T-SPOT for improving classification in MTB infection. Importantly, the detection of lymphocyte non-specific function not only plays a complementary diagnostic role, but may also provide advantages if further developed as an approach for immune monitoring and management in TB patients. Regarding that the numerous challenges still present in combating TB and critical need for better tools, our novel and adaptable lymphocyte non-specific function detection may support ongoing efforts in eliminating TB globally. The early diagnosis and guided initiation of anti-TB treatment brought by the present diagnostic model would help reduce transmission and mortality of the disease.

## Data Availability Statement

The original contributions presented in the study are included in the article/**Supplementary Material**. Further inquiries can be directed to the corresponding authors.

## Ethics Statement

The studies involving human participants were reviewed and approved by the committee of Tongji hospital, Tongji Medical College, Huazhong University of Science and Technology. The patients/participants provided their written informed consent to participate in this study.

## Author Contributions

YL, YX, WYL, SW, ZS, and FW designed and oversaw the study. QL and GT contributed to lymphocyte non-specific function assay. HS, WL, and LM conducted T-SPOT assay. XY and YZ coordinated data collection and management. YL and YC did the statistical analysis. YL wrote the first manuscript draft. All authors contributed to the article and approved the submitted version.

## Funding

This work was funded by the National Natural Science Foundation (grant number 81401639 and 81902132) and the National Mega Project on Major Infectious Disease Prevention of China (grant number 2017ZX10103005-007).

## Conflict of Interest

The authors declare that the research was conducted in the absence of any commercial or financial relationships that could be construed as a potential conflict of interest.

## Supplementary Material

Supplementary Figure 1 The flow analysis template of lymphocyte non-specific function assay. Diluted whole blood was stimulated with Leukocyte Activation Cocktail for 4 h. Representative flow plots showing the gating strategies of IFN-γ^+^ cells in CD4^+^, CD8^+^ T cells, and NK cells.

Supplementary Figure 2 The correlation between ESAT-6 spot number and CFP-10 spot number. Scatter plot showing the correlation between ESAT-6 spot number and CFP-10 spot number. ESAT-6, early secreted antigenic target 6; CFP-10, culture filtrate protein 10.

Supplementary Figure 3 The performance of the diagnostic model incorporating ESAT-6 spot number in distinguishing ATB patients from LTBI individuals. Scatter plots showing the predictive value of diagnostic model incorporating ESAT-6 spot number in ATB patients (n=49) and LTBI individuals (n=64). Bars indicated the medians and interquartile ranges. ****P*<0.001 (Mann-Whitney *U* test). Blue dotted line indicates the cutoff value in distinguishing these two groups. ROC analysis showing the performance of diagnostic model incorporating ESAT-6 spot number in discriminating ATB patients from LTBI individuals.
