# Supplementary figures and images for: Lymphocyte Non-Specific Function Detection Facilitating the Stratification of Mycobacterium tuberculosis Infection
Source: Front Immunol. 2021 Apr 19;12:641378. doi: 10.3389/fimmu.2021.641378 (PMC8092189; doi:10.3389/fimmu.2021.641378)

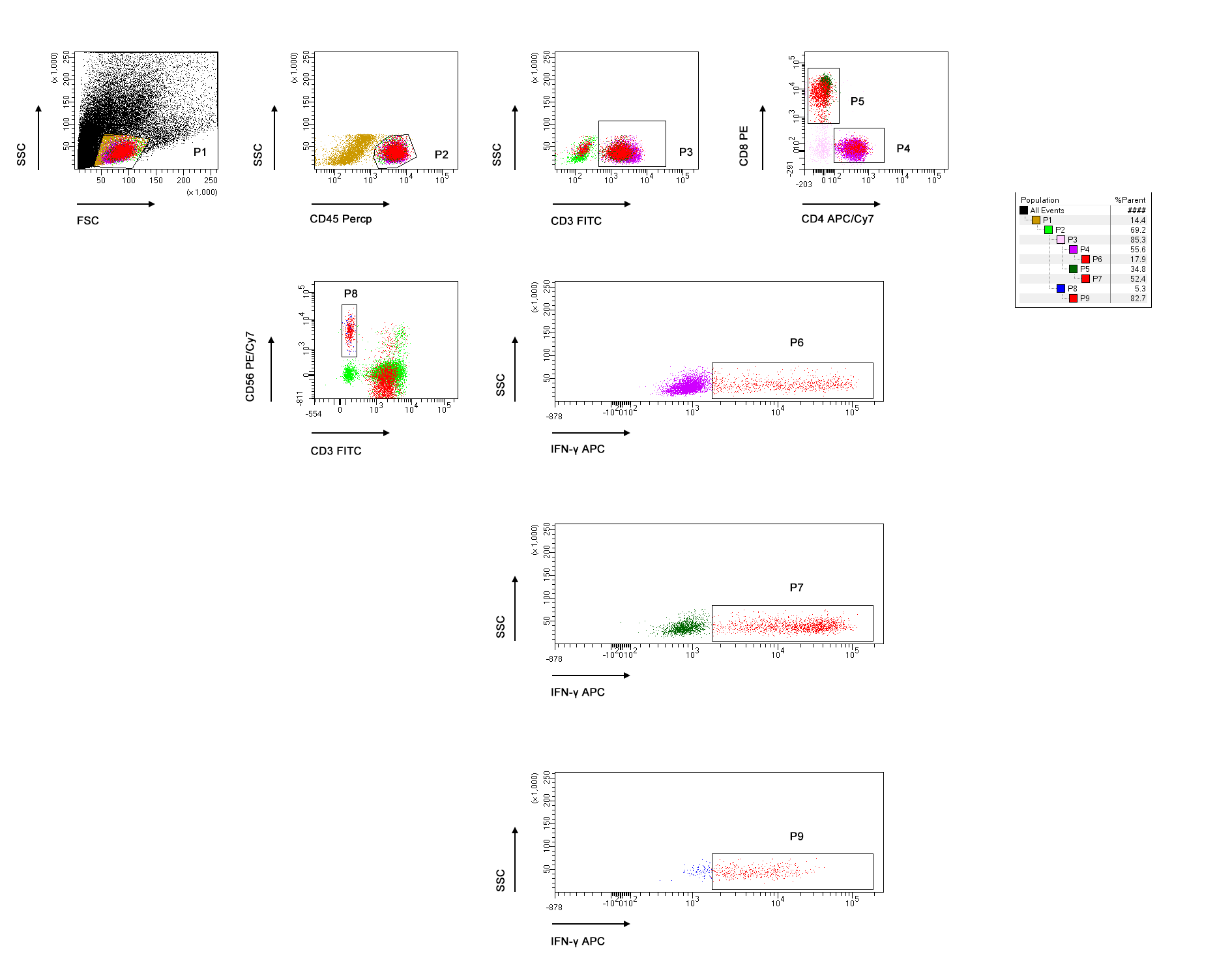

Supplement: Supplementary Figure 1 — The flow analysis template of lymphocyte non-specific function assay. Diluted whole blood was stimulated with Leukocyte Activation Cocktail for 4 h. Representative flow plots showing the gating strategies of IFN-γ+ cells in CD4+, CD8+ T cells, and NK cells. [file Image_1.tif]

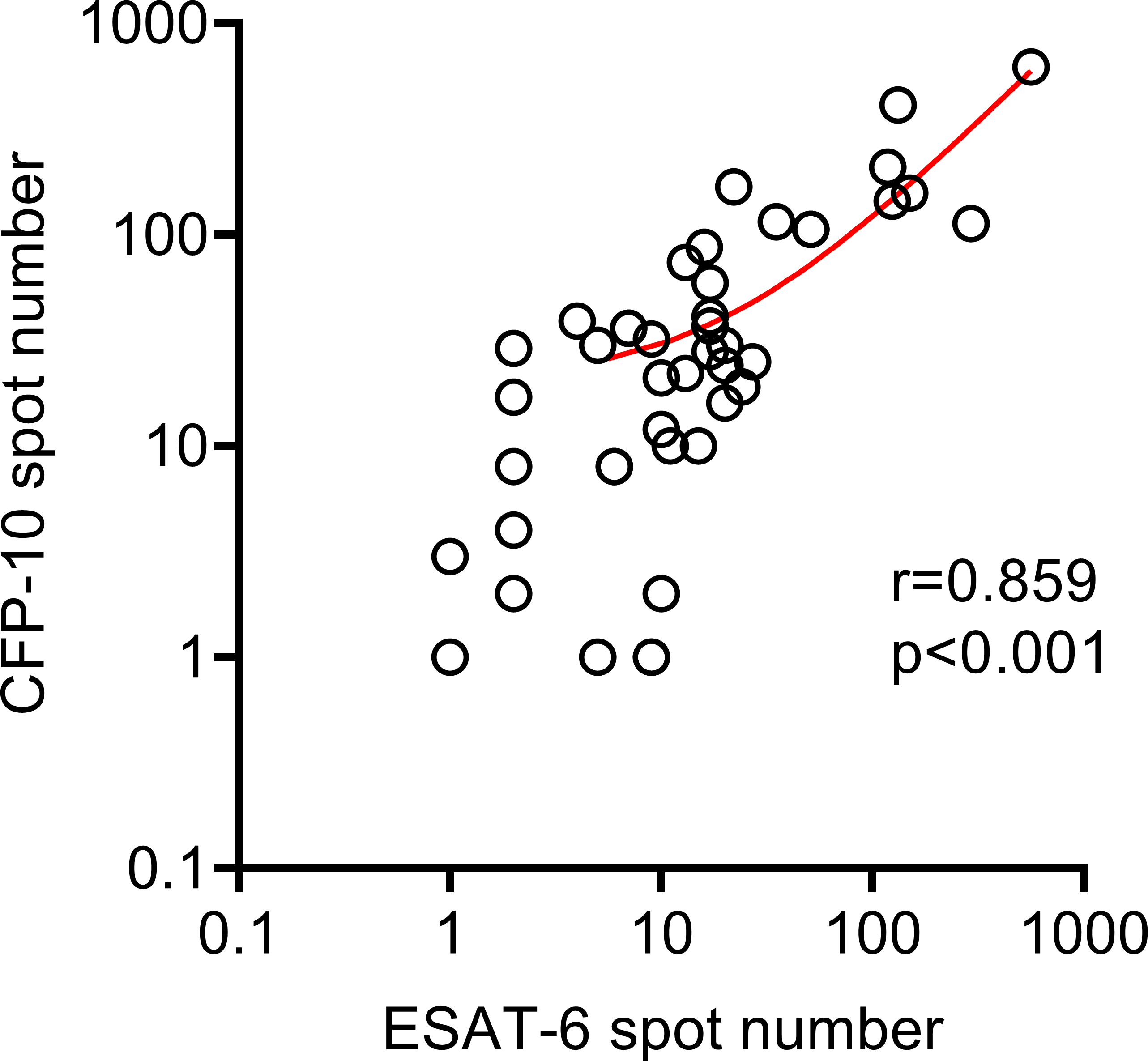

Supplement: Supplementary Figure 2 — The correlation between ESAT-6 spot number and CFP-10 spot number. Scatter plot showing the correlation between ESAT-6 spot number and CFP-10 spot number. ESAT-6, early secreted antigenic target 6; CFP-10, culture filtrate protein 10. [file Image_2.tif]

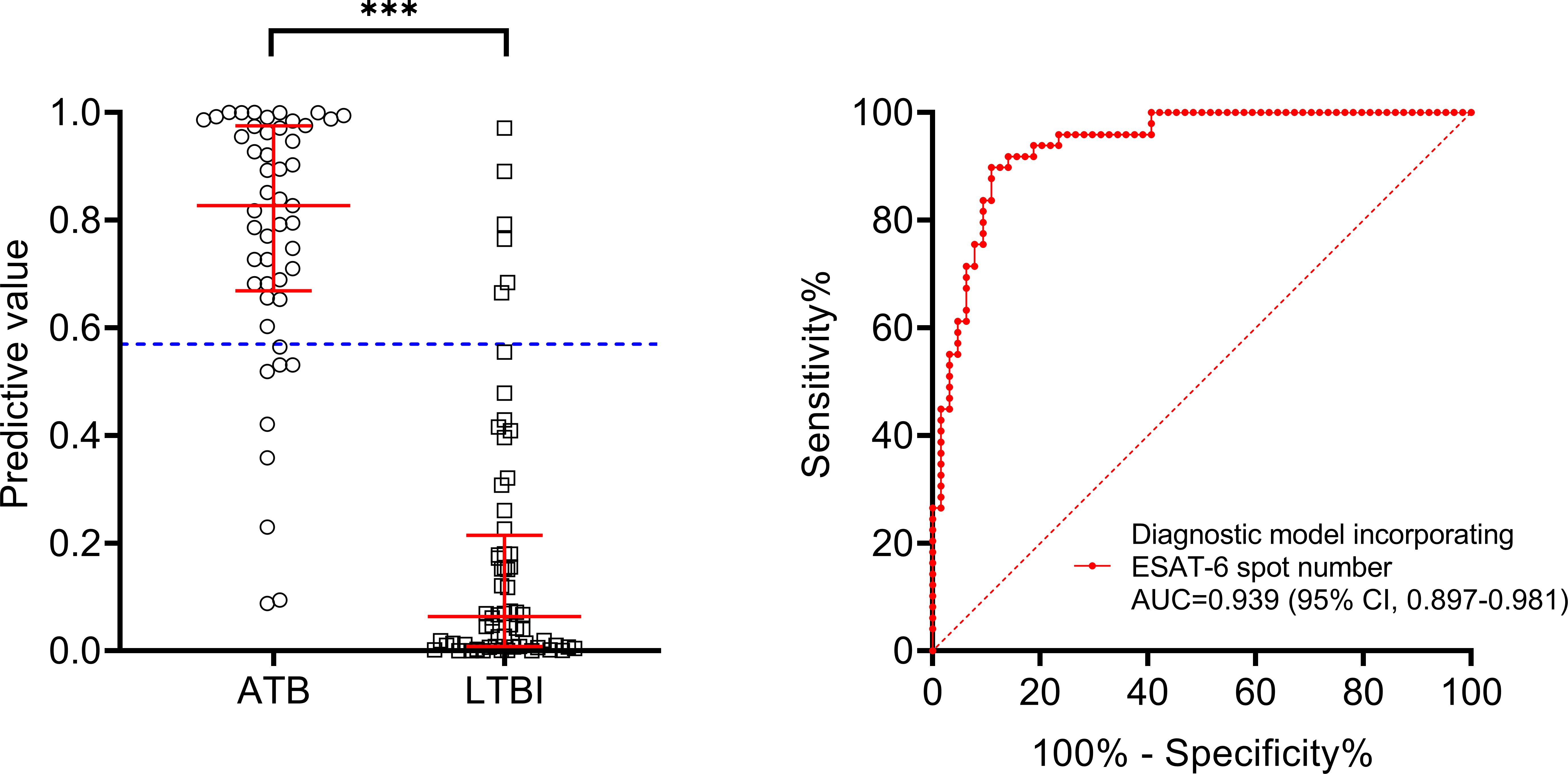

Supplement: Supplementary Figure 3 — The performance of the diagnostic model incorporating ESAT-6 spot number in distinguishing ATB patients from LTBI individuals. Scatter plots showing the predictive value of diagnostic model incorporating ESAT-6 spot number in ATB patients (n=49) and LTBI individuals (n=64). Bars indicated the medians and interquartile ranges. ***P<0.001 (Mann-Whitney U test). Blue dotted line indicates the cutoff value in distinguishing these two groups. ROC analysis showing the performance of diagnostic model incorporating ESAT-6 spot number in discriminating ATB patients from LTBI individuals. [file Image_3.tif]
